# Antithyroglobulin and Antiperoxidase Antibodies Can Negatively Influence Pregnancy Outcomes by Disturbing the Placentation Process and Triggering an Imbalance in Placental Angiogenic Factors

**DOI:** 10.3390/biomedicines12112628

**Published:** 2024-11-17

**Authors:** Kamila Tańska, Piotr Glinicki, Beata Rebizant, Piotr Dudek, Wojciech Zgliczyński, Małgorzata Gietka-Czernel

**Affiliations:** 1 Department of Endocrinology, Centre of Postgraduate Medical Education, 01-813 Warsaw, Poland; pldudek@wp.pl (P.D.); wojciech.zgliczynski@cmkp.edu.pl (W.Z.);; 2 EndoLab Laboratory, Centre of Postgraduate Medical Education, 01-813 Warsaw, Poland; 3 Department of Obstetrics, Perinatology and Neonatology, Centre of Postgraduate Medical Education, 01-813 Warsaw, Poland; bnppn@cmkp.edu.pl

**Keywords:** thyroid antibodies, placental growth factor, soluble vascular endothelial growth factor receptor 1, soluble endoglin, gestational hypertension, preeclampsia

## Abstract

**Background/Objectives**: Thyroid autoimmunity (TAI) affects about 15% of women of reproductive age and can negatively affect pregnancy outcomes. One possible mechanism for pregnancy complications can be attributed to a disturbed process of placentation caused by thyroid antibodies. To test this hypothesis, placental hormones and angiogenic factors in pregnant women with TAI were evaluated. **Methods**: Fifty-eight hypothyroid women positive for TPOAb/TgAb, thirty-three hypothyroid women negative for TPOAb/TgAb, and thirty-nine healthy controls were enrolled in this study. Maternal thyroid function tests were established every month throughout pregnancy, and angiogenic placental factors, pro-angiogenic placental growth factor (PlGF); two anti-angiogenic factors, soluble vascular endothelial growth factor receptor 1 (sFlt-1) and soluble endoglin (sEng); and placental hormones, estradiol, progesterone, and hCG, were determined during each trimester. **Results**: Obstetrical and neonatal outcomes did not differ between the groups. However, several detrimental effects of thyroid antibodies were observed. These included a positive correlation between TgAb and the sEng/PlGF ratio in the first trimester and positive correlations between TPOAb and sFlt-1 and between TgAb and the sFlt-1/PlGF ratio in the third trimester. TgAbs in the first trimester was a risk factor for gestational hypertension and preeclampsia. **Conclusions**: Our study indicates that TPOAbs and TgAbs can exert a direct harmful effect on placentation, leading to disturbances in the production of placental angiogenic factors and, consequently, to an increased risk of gestational hypertension and preeclampsia.

## 1. Introduction

Thyroid autoimmunity (TAI) is the most commonly reported autoimmune disorder in women of reproductive age [1,2,3]. The presence of circulating anti-thyroid antibodies targeting thyroid peroxidase (TPOAb) and thyroglobulin (TgAb), even in the setting of euthyroidism, is a well-recognized risk factor for adverse outcomes in pregnancy [4,5]. TAI has been proven to be associated with an increased risk of spontaneous miscarriage [6,7,8], preterm birth [9,10,11], and recurrent pregnancy loss [12,13]. It has also been suggested to be linked with an increased risk of preeclampsia, placenta previa, polyhydramnios, placental abruption, premature rupture of membranes, and gestational diabetes mellitus [4,14,15]. Two possible mechanisms may account for these maternal obstetrical complications: generalized immune dysregulation with TAI as an epiphenomenon and thyroid hormone deficiency.

TAI can be accompanied by either organ-specific autoantibodies (autoimmune polyglandular syndromes 1–4 with anti-ovarian antibodies) or non-organ-specific antibodies (systemic lupus erythematosus, Sjögren’s syndrome, and rheumatoid arthritis with antinuclear, anti-dsDNA, anti-ssDNA, antiphospholipid, and anti-laminin-1 antibodies), which can both exert a negative impact on female fertility and reproductive function [16,17]. Additionally, TAI can itself induce an inhibition of immune tolerance, causing a systemic imbalance of helper lymphocyte Th1/Th2/Th17 and Treg activity and triggering increased secretion of several inflammatory cytokines (Il-2, IL-17, INF-γ) [18].

According to multiple reports, euthyroid women with TPOAb and/or TgAb positivity have higher serum TSH compared with healthy controls (although within the normal range) [19,20]. TAI also constitutes a risk of subclinical hypothyroidism development throughout gestation. In 1994, Glinoer et al. observed that 20% of euthyroid women with TPOAb or TgAb positivity developed subclinical hypothyroidism during pregnancy [21]. Since then, other authors have reported this complication in 19–40% of cases [22,23]. Korevaar et al. demonstrated that TPOAb-positive euthyroid women displayed an impaired thyroidal response to human chorionic gonadotropin (hCG) [24]. Hou et al. reported a similar attenuated response of fT4 production following hCG stimulation in TgAb-positive women [25]. Most recently, an individual participant data meta-analysis by Bliddal et al. reported an increase in TSH in euthyroid pregnant women with isolated TPOAb positivity or TgAb positivity, which was amplified in individuals positive for both antibodies [26]. Thus, the 2017 Guidelines of the American Thyroid Association recommend the measurement of serum TSH concentration every four weeks throughout mid-pregnancy in euthyroid pregnant women who are TPOAb- or TgAb-positive [27]. This strategy enables the early recognition of subclinical hypothyroidism and initiation of levothyroxine (LT4) therapy to prevent the harmful consequences of thyroid hormone deficiency. However, numerous randomized trials have failed to achieve higher rates of pregnancies or live births after LT4 treatment of euthyroid TAI women with subfertility or recurrent miscarriages [28,29,30].

Several studies provide evidence that thyroid-specific genes, including genes for the sodium/iodide symporter, pendrin, TPO, and Tg, are expressed in luteal phase endometrium, syncytiotrophoblast, and invasive trophoblast. Rahnama et al. have provided evidence that TPO is also expressed at the protein level in these tissues [31]. These observations are consistent with the hypothesis that an interaction between thyroid antibodies and reproductive tissues expressing thyroid proteins may occur and lead to reproductive tissue damage. This is another potential explanation for the higher risk of subfertility and negative pregnancy outcomes in women with TAI.

Normal pregnancy development depends on immune tolerance to the semi-allogeneic fetus, successful invasion of the maternal tissues by extravillous trophoblast, and efficient remodeling of the spiral arteries to ensure adequate blood supply to the developing embryo and placenta. The uterine natural killer cells (uNK) are thought to play a major role in the latter process, being the main source of angiogenic growth factors [32]. Several angiogenic factors have been studied to evaluate the placentation process and placenta function. The two most clinically useful biomarkers are placental growth factor (PlGF) and soluble FMS-like tyrosine kinase-1 (sFlt-1; or soluble vascular endothelial growth factor [VEGF] receptor 1) [33,34,35]. PlGF is a pro-angiogenic factor belonging to the vascular endothelial growth factor (VEGF) family, whereas sFlt-1 is a potent soluble antagonist of VEGF and PlGF signaling. An unbalanced sFlt-1/PlGF ratio (with high sFlt-1 levels and low PlGF levels) has been associated with negative pregnancy outcomes, such as preeclampsia, fetal growth restriction (FGR), and small for gestational age (SGA) newborn babies [36,37]. Soluble endoglin (sEng), a transforming growth factor-beta (TGF-β) co-receptor, is another biomarker of placental origin with anti-angiogenic properties. Specifically, sEng has been demonstrated to dysregulate signaling between TGF-β and endothelial nitric oxide synthase. Elevated sEng was found to be associated with a more severe course of preeclampsia and FGR [38].

In the current study, our aim was to evaluate whether TPOAb and TgAb positivity disturb placental hormonal function and placental angiogenic factors in women with TAI. For this purpose, we compared serum concentrations of selected placental hormones and angiogenic factors, the hemodynamic features of the placenta, and pregnancy outcomes, among hypothyroid women who were positive for TPOAb and/or TgAb, hypothyroid subjects who were negative for TPOAb/TgAb, and healthy controls.

## 2. Materials and Methods

### 2.1. Patient Cohort

In total, 125 hypothyroid pregnant women with autoimmune thyroiditis who visited the Outpatient Department of Endocrinology, Centre of Postgraduate Medical Education (Warsaw, Poland), between October 2018 and December 2023 were recruited for this study. Inclusion criteria were as follows: (1). written consent to participate in this study; (2). age between 18 and 40 years; (3). singleton pregnancy; (4). gestational age ≤ 15 weeks; and (5). TPOAb or TgAb positivity or ultrasonographic features typical for autoimmune thyroiditis. The exclusion criteria included (1). refusal to participate in this study; (2). multiple pregnancy; (3). chronic illnesses other than hypothyroidism; (4). treatment with drugs known to interfere with thyroid function (except LT4); and (5). a previous history of infertility treatment, pregnancy loss, or obstetrical complications. Of the 125 patients recruited, 34 participants were eliminated from this study due to incomplete laboratory data (n = 21) or because they were lost to follow-up (n = 13). Because our study was conducted during the COVID-19 pandemic, many of the eliminated participants chose to avoid direct contact with healthcare institutions while pregnant. Ultimately, ninety-one hypothyroid pregnant women with autoimmune thyroiditis were included in our final analysis.

The patients were divided into two groups: Group 1 (n = 58; hypothyroid, positive for TPOAb/TgAb) and Group 2 (n = 33; hypothyroid, negative for TPOAb/TgAb). As a control (Group 3; n = 39), we recruited healthy euthyroid pregnant women from the Outpatient Department of Obstetrics, Perinatology, and Neonatology, Centre of Postgraduate Medical Education, who were negative for TPOAb and TgAb and whose thyroid sonograms exhibited no features of autoimmune thyroiditis.

In Group 1 and Group 2, hypothyroidism was recognized before gestation or during the first trimester of pregnancy. A diagnosis of hypothyroidism in the pre-conception period was based on the same criteria as those adopted for the general population. Until 2021, first-trimester pregnancy hypothyroidism was diagnosed with a TSH concentration equal to or greater than 2.5 mIU/L (in line with contemporary recommendations) [39,40,41]. From 2021 onward, a TSH concentration above 3.18 mIU/L was considered abnormal based on the results of a Polish multicenter study [42]. All women who were diagnosed as hypothyroid after becoming pregnant presented with subclinical disease. During pre-conception and pregnancy, the dose of LT4 was adjusted to achieve a TSH concentration below 2.5 mIU/L.

This study was approved by the institutional review board (internal protocol N 42/PB/2018), and written informed consent was obtained from all participants.

### 2.2. Study Design

This was an observational prospective single-center study. Hypothyroid women were followed every 4 weeks throughout pregnancy. Serum TSH, fT4, and fT3 concentrations were determined before each visit, and the LT4 dose was titrated to achieve a target TSH value. Additionally, C-reactive protein (CRP), angiogenic placental factors (PlGF, sFlt-1, and sEng), and placental hormones (estradiol (E2), progesterone (P) and beta-human chorionic gonadotropin (β-hCG)) were measured during each trimester. Standard ultrasound evaluations were performed at 11–14 weeks, 18–22 weeks, and 28–32 weeks. In the control group, clinical assessments and the abovementioned biochemical, hormonal, and ultrasound measurements were performed once during each trimester. Gestational hypertension and preeclampsia were diagnosed according to established criteria [43,44].

### 2.3. Laboratory Analyses

Blood samples were taken in the morning (7.00 a.m. to 10.00 a.m.) after an overnight fast using a standard puncture of a cubital vein. Serum TSH, fT4, fT3, TPOAb (normal range ≤ 60 IU/mL), and TgAb (normal range ≤ 60 IU/mL) were determined with a chemiluminescence immunoassay (CLIA, Washington, DC, USA) using an ADVIA Centaur XPT analyzer (Siemens, Munich, Germany). Serum E2 and P were obtained using chemiluminescence methods (CLIA) (Liaison^®^, DiaSorin, Saluggia, Italy). Serum β-hCG was measured using an immunochemical technique (Cobas 6000 and Cobas E411, Roche Diagnostics, Indianapolis, IN, USA), and serum CRP (normal range 0–5 mg/L) was determined using the immunoturbidimetric method (Cobas 6000 and Cobas E411, Roche Diagnostics, USA). To determine placental angiogenic factors, venous blood samples were drawn into EDTA2K collection tubes. The tubes were then centrifuged (10 min, 3500 rpm) to separate the plasma, and this was stored at −80 °C until assay. Human PlGF was determined using a specific ELISA kit (R&D Systems^®^, Inc., Minneapolis, MN, USA): sensitivity: 7.0 pg/mL; assay range: 0.0–1000.0 pg/mL; intra-assay and inter-assay precision: 3.6% and 11.0%, respectively. Human sFlt-1 was also measured using a specific ELISA kit (R&D Systems^®^, Inc., USA): sensitivity: 4.17 pg/mL; assay range: 0.0–2000.0 pg/mL; intra-assay and inter-assay precision: 2.4% and 6.3%, respectively. Finally, human sEng/CD105 was also measured using a specific ELISA kit (R&D Systems^®^, Inc., USA): sensitivity: 0.007 ng/mL; assay range: 0.0–10.0 ng/mL; intra-assay and inter-assay precision: 3.2% and 6.5%, respectively.

### 2.4. Ultrasound Measurements

Ultrasound evaluations were performed using a Voluson E8 Expert GE scanner with an abdominal convex transducer RAB6-D and C2-9-D. Examinations between 11 and 14 weeks were conducted in accordance with the recommendations of the Fetal Medicine Foundation (FMF). The risks of FGR and preeclampsia were calculated according to FMF protocol in all patients. During ultrasound examinations at more than 14 weeks of pregnancy, the following biometric parameters of the fetus were assessed: biparietal diameter (BPD), head circumference (HC), abdominal circumference (AC), femur length (FL), humerus length (HL). The Hadlock 2 formula [Log 10 (weight) = 1.335 − 0.0034*AC*FL + 0.0316*BPD + 0.0457*AC + 0.1623*FL] and Hadlock centile charts were used to calculate the estimated fetal weight [45]. Fetuses with an estimated weight above the 90th percentile were classified as large for gestational age (LGA). The criteria for diagnosing SGA/FGR fetuses were used according to the Delphi consensus [46]. Pulsatility index in the umbilical artery and uterine arteries was assessed using a pulsed Doppler gate. The parameters were plotted on centile charts built into Sonomedica (v.1.7.0) or Astraia (v.29.0.0) software (depending on the time of examination).

### 2.5. Statistical Analyses

All statistical analyses were performed using statistical software R (version 4.1.2). For numeric parameters, the following basic descriptive statistics were used: mean, standard deviation, median, 1st quartile, 3rd quartile, and range. The normality of a distribution was checked using the Shapiro–Wilk test and additionally verified with skewness and kurtosis.

For comparisons of nominal parameters between groups, the following statistical tests were used: Pearson’s chi-squared test in cases satisfying the assumption of the expected number of observations and Fisher’s exact test in cases not satisfying the assumption of the expected number of observations.

For comparisons of numeric parameters between groups, the following statistical tests were used: an ANOVA with Tukey for pairwise comparisons in cases with a normal distribution of the parameter in each of the three groups and a Kruskal–Wallis test with Dunn and Bonferroni adjustment for pairwise comparisons in cases for which the assumption on normality was not satisfied.

All correlations were measured with the Spearman rank correlation coefficients. Univariate and multivariable logistic regression models were estimated to evaluate the dependence of selected pregnancy complications, including miscarriage, preterm birth, cervical insufficiency, pregnancy-induced hypertension, and preeclampsia, on various independent variables. The clinical and laboratory parameters analyzed as independent variables were as follows: age, BMI, weight gain, parity (multiparous vs. primiparous), TSH, fT4, TPOAb, TgAb, PlGF, sEng, sFlt-1, sEng/PlGF, and sFlt-1/PlGF. To ensure a realistic interpretation of odds ratios, units for some of the parameters were transformed. Those parameters were sFlt-1 (divided by 1000), TPOAb and TgAb (divided by 10); sEng/PlGF, and sFlt-1/PlGF (divided by 100). Each logistic regression analysis was performed separately for each trimester.

Finally, a receiver operating characteristics (ROC) analysis was used to identify the optimal cut-offs of selected parameters to predict pregnancy complications. Optimal cut-offs were indicated with the Youden method. The area under the curve (AUC) was calculated to assess general predictive quality. The sensitivity, specificity, accuracy, positive predictive value (PPV), and negative predictive value (NPV) were calculated for those cut-off points. A *p* value of <0.05 was used to assess statistical significance.

## 3. Results

### 3.1. Characteristics of Study Groups

The characteristics of the participants are presented in Table 1. The three analyzed groups did not differ with respect to age, BMI, educational level, smoking habits, parity, and weeks of gestation at recruitment. None of the participants declared any alcohol use during pregnancy. In Group 1, at enrollment, 57/58 women (98.3%) were TPOAb-positive, 12/58 were TgAb-positive (20.7%), and 11/58 participants (18.9%) were positive for both antibodies. In the second and third trimesters, respectively, 46/51 (90%) and 40/48 (83.3%) women remained TPOAb-positive, and 7/51 (13.7%) and 5/48 (10.4%) still tested positive for TgAb.

### 3.2. Pregnancy and Neonatal Outcomes

There were no statistically significant differences in negative pregnancy outcomes—spontaneous miscarriage, preterm birth, cervical insufficiency, cesarean section, gestational diabetes, gestational hypertension, and preeclampsia—between the groups (Table 2). However, the real rate of spontaneous miscarriage remained uncertain due to the adopted inclusion criteria (enrollment ≤ 15 gestational weeks). Surprisingly, a relatively high rate of pregnancy complications was noted among control participants: preterm births, 12.8%; gestational diabetes, 25.6%; gestational hypertension, 10.3%. Of note, a high rate of cesarean sections among women in Group 1 was observed (43.8%), which was twice as common as in controls. The statuses of newborns with respect to birth weight, FGR/SGA, Apgar score, and necessity of *neonatal intensive care unit* admission (*NICU*) did not differ among the analyzed groups. To evaluate whether TSH concentration in TPOAb-/TgAb-positive women influenced a pregnancy course, Group 1 was split into subgroups depending on baseline TSH concentration: a subgroup with TSH < 2.5 mIU/L and a subgroup with TSH ≥ 2.5 mIU/L (Table S1), as well as a subgroup with TSH < 3.0 mIU/L and a subgroup with TSH ≥ 3.0 mIU/L (Table S2). The risk of pregnancy and neonatal complications in TPOAb-/TgAb-positive women with TSH ≥ 2.5 mIU/L and TSH ≥ 3.0 mIU/L was not higher than in hypothyroid antibody-negative women and controls.

### 3.3. Laboratory Results

A comparison of TSH, free thyroid hormones, and CRP concentrations among study groups is presented in Table 1. TSH concentration was higher in hypothyroid Groups 1 and 2 in the first trimester, did not differ between groups in the second trimester, and was lowest in Group 1 in the third trimester. FT4 concentration did not differ between groups in the first trimester but was higher in hypothyroid Groups 1 and 2 (compared with the control group) over the next two trimesters. FT3 concentration differentiated the groups only in the first trimester, where it was lower in hypothyroid Groups 1 and 2 (compared with the control group). CRP did not differentiate the groups in any statistically significant way (*p* > 0.05 in all three trimesters).

Data on the concentrations of placental hormones and growth factors in the analyzed groups throughout pregnancy are summarized in Table 3. Additionally, the sFlt-1/PlGF and sEng/PlGF ratios were determined to find an imbalance between pro- and anti-angiogenic placental factors. TPOAb-/TgAb-positive hypothyroid pregnant women had lower E2 concentrations during the first trimester than antibody-negative hypothyroid pregnant women: 1682.00 vs. 2440.00 pg/mL (*p* adj = 0.016). In Group 1, the P concentration during the second trimester was also lower (compared with the control group): 37.00 vs. 50.25 ng/mL (*p* adj = 0.003). No significant differences in β-hCG concentration were observed among the groups. Of the analyzed placental growth factors, only sEng concentrations differentiated the groups. The anti-angiogenic factor sEng was significantly higher in TPOAb-/TgAb-positive hypothyroid pregnant women than in antibody-negative hypothyroid pregnant women during all three trimesters (*p* adj = 0.026, *p* adj = 0.022, and *p* adj = 0.003, respectively).

### 3.4. Placental Hemodynamics

The placental hemodynamics parameters did not differentiate the groups in any statistically significant way (*p* > 0.05) (see Table S3).

### 3.5. Correlations Between TPOAbs/TgAbs and Placental Hormones and Angiogenic Factors and Hemodynamic Parameters

In hypothyroid pregnant women from Group 1, a positive correlation was observed between TPOAbs and anti-angiogenic sFlt-1 during the third trimester (rho = 0.31; *p* = 0.040). TgAbs were negatively correlated with pro-angiogenic PlGF during the first trimester and E2 during the third trimester (rho = −0.27, *p* = 0.037 and rho = −0.29, *p* = 0.043, respectively). There was also a positive correlation between TgAbs and the sEng/PlGF ratio during the first trimester and between TgAbs and the sFlt-1/PlGF ratio during the third trimester (rho = 0.28, *p* = 0.034 and rho = 0.30, *p* = 0.049, respectively).

No correlation between TPOAbs/TgAbs and any of the hemodynamic placental parameters was observed (Table 4).

### 3.6. Logistic Regression Analyses for Selected Pregnancy Complications

The outcomes of the univariate logistic regression models focusing on each predictor separately, including miscarriage, preterm birth, cervical insufficiency, gestational hypertension, and preeclampsia are presented in the Supplementary Materials (Table S4).

From among selected clinical (age, BMI, weight gain, and parity) and biochemical factors (TSH, fT4, TPOAb, TgAb, PlGF, sEng, sFlt-1, sEng/PlGF, and sFlt-1/PlGF), no single factor turned out to have a significant impact on the odds of miscarriage in either the first or second trimester (*p* > 0.05).

No factor appeared to have a significant impact on the odds of preterm birth in either the first or second trimester (*p* > 0.05). In the third trimester, the concentrations of sEng and sFlt-1 determined the risk of preterm birth: OR 1.42 CI95 (1.14;1.91), *p* = 0.006, and OR 1.22 CI95 (1.08;1.41), *p* = 0.002, respectively.

The risk of gestational hypertension was determined by TgAb concentration measured in the first trimester: OR 1.09 CI95 [1.01;1.20], *p* = 0.024. In the second trimester, the odds were impacted by sEng and sEng/PlGF: OR 1.68 CI95 (1.09;2.67), *p* = 0.020, and OR 4.65 CI95 (0.92;21.65), *p* = 0.048, respectively. In the third trimester, the risk of gestational hypertension was determined by the levels of PlGF, sEng, sFlt-1, and sFlt-1/PlGF. The highest risk was related to the sFlt-1/PlGF ratio: OR 9.77 CI95 (2.67;54.82), *p* = 0.003.

The risk of preeclampsia was determined by TgAb concentration measured in the first trimester: OR 1.13 CI95 (1.04;1.27), *p* = 0.007. In the second trimester, the odds were impacted by sEng, sEng/PlGF, and sFlt-1/PlGF: OR 2.23 CI95 (1.17;5.19), *p* = 0.025, OR 8.23 CI95 (0.90;78.98), *p* = 0.048, and OR 5.30 CI95 (0.84;29.37), *p* = 0.047, respectively. In the third trimester, the risk of preeclampsia was determined by the ratio of sFlt-1/PlGF: OR 9.74 CI95 (2.21;152.09), *p* = 0.018.

Outcomes of multivariate logistic regression models for analyses focusing on each predictor separately are presented in Table 5.

In the first trimester, the concentration of TSH had a positive impact on the odds of miscarriage: OR 1.22 CI95 (1.03;1.65), *p* = 0.045. The logistic regression multivariate model for the second trimester did not accomplish sufficient outcomes.

No factor turned out to have a significant impact on the odds of preterm birth in the first trimester (*p* > 0.05). In the second trimester, the concentration of sEng and, in the third trimester, the concentration of sFlt-1 had a positive impact on the odds of preterm birth: OR 1.45 CI95 (1.00;2.13), *p* = 0.046, and OR = 1.28 CI95 [1.05;1.65], *p* = 0.025, respectively.

No predictor measured in the first trimester included in the multivariate model for risk of gestational hypertension turned out to be significant (*p* > 0.05). The risk of gestational hypertension was determined by the concentration of sEng measured in the second trimester: OR 1.91 CI95 (1.16;3.48), *p* = 0.016. In the third trimester, the risk of gestational hypertension was impacted by the levels of sEng and the sFlt-1/PlGF ratio: OR 1.37 CI95 (1.05;1.91), *p* = 0.028, and OR 7.49 CI95 (1.57;71.74), *p* = 0.039, respectively.

The risk of preeclampsia was determined by the TgAb concentration measured in the first trimester: OR 1.13 CI95 (1.04;1.27), *p* = 0.007. In the second trimester, the odds were impacted by the sEng/PlGF ratio: OR 19.85 CI95 (1.36;879.73), *p* = 0.040. The logistic regression multivariate model for the third trimester did not accomplish sufficient outcomes.

### 3.7. ROC Analyses for Selected Pregnancy Complications

An ROC analysis was performed to assess the effectiveness of selected parameters in predicting miscarriage, preterm birth, cervical insufficiency, and gestational hypertension. All outcomes are summarized in Table 6. ROC analyses were not performed for preeclampsia because of the small sample size.

**Figure 1:**
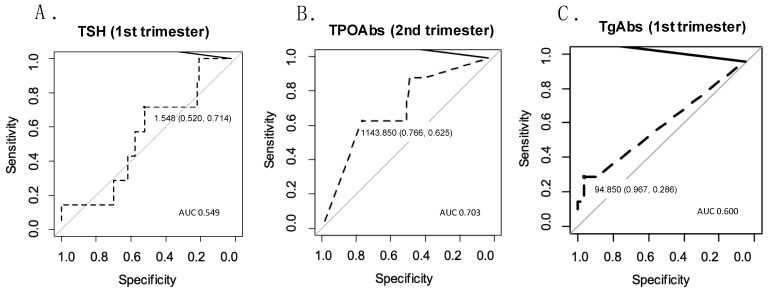
ROC curves for predicting obstetrical complications based on selected parameters. (**A**) ROC curve for predicting miscarriage based on TSH measured in the first trimester; (**B**) ROC curve for predicting cervical insufficiency based on TPOAbs measured in the second trimester; (**C**) ROC curve for predicting gestational hypertension based on TgAbs measured in the first trimester.

Predictive qualities for miscarriage were verified for TSH measured in the first trimester. The area under the curve was AUC = 0.549 CI95 [0.357;0.745], *p* = 0.037, which indicated a low predictive quality. The optimal cut-off for TSH was 1.55, with a related sensitivity of 71% and specificity of 52%. The ROC curve for TSH measured in the first trimester is presented in Figure 1.

The preterm birth statistical significance of the ROC curve was identified for sEng and sFlt-1, both of which were measured in the third trimester. Better predictive quality was identified in the case of sFlt-1: AUC = 0.796 CI95 (0.601;0.915), *p* = 0.002). For sEng, the area under the curve was AUC = 0.698 CI95 (0.471;0.879), *p* = 0.001.

TPOAbs measured in the second trimester, sEng/PlGF measured in the first trimester, and sFlt-1/PlGF measured in the first trimester were analyzed as potential predictors of cervical insufficiency. The highest predictive quality was identified for TPOAb: AUC = 0.703 CI95 (0.504;0.863), *p* = 0.038). The optimal cut-off was 1143.85 IU/mL, with a sensitivity of 62% and a specificity of 77% (Figure 1).

The effectiveness of predicting gestational hypertension was high for the sFlt-1/PlGF ratio and PlGF, both of which were measured in the third trimester: AUC = 0.882 CI95 (0.702;0.988), *p* < 0.001, and AUC = 0.857 CI95 (0.733;0.957), *p* = 0.002, respectively. Other parameters with significant ROC curves were sFlt-1 measured in the third trimester (AUC = 0.797 CI95 [0.486;0.996], *p* < 0.001), sEng measured in the second and third trimesters (AUC = 0.778 CI95 [0.599;0.924], *p* = 0.001, and AUC = 0.765 CI95 [0.615;0.904], *p* = 0.019, respectively), and TgAbs measured in the first trimester (AUC = 0.600 CI95 [0.439;0.768], *p* = 0.026). The optimal cut-off of TgAb was 94.85 IU/mL, with a sensitivity of 29% and a specificity of 97% (Figure 1).

## 4. Discussion

The aim of this study was to prospectively evaluate the influence of TPOAbs and/or TgAbs on placental hormonal and angiogenic function, irrespective of TSH and thyroid hormone concentrations. To accomplish this, we enrolled pregnant women who were either already being treated for or were newly diagnosed with hypothyroidism. Such inclusion criteria allowed us to maintain the relative homogeneity of the study group. If only euthyroid TAI women were recruited, we would expect that 20–40% of the subjects would develop hypothyroidism over the course of pregnancy. It has previously been observed that hypothyroid patients receiving replacement therapy with LT4 had non-physiological thyroid hormone levels, even after achieving an optimal TSH concentration, i.e., higher fT4 and lower fT3 concentrations in comparison to euthyroid subjects [47,48]. To account for this, we matched our study group with both healthy controls and hypothyroid women who were TPOAb-/TgAb-negative. In our small study, we showed that pregnancy outcomes in strictly controlled hypothyroid women, irrespective of TPOAb/TgAb status, were similar to those in healthy controls. Some of our patients were diagnosed with subclinical hypothyroidism (SH) in the first trimester of pregnancy based on a TSH concentration equal to or greater than 2.5 mIU/L. Although several authors have reported that LT4 treatment for SH is especially effective in the prevention of pregnancy loss when the pretreatment TSH concentration is above 4.0 mIU/L, this was not the case for our cohort [49,50].

Because our control group exhibited an unexpectedly high rate of maternal pregnancy complications, which could influence the obtained results, we further evaluated the possible risk factors for selected adverse pregnancy outcomes—specifically, miscarriage, preterm birth, cervical insufficiency, gestational hypertension, and preeclampsia. We excluded cesarean section from the obstetrical complications considered here because surgical delivery can be overused in our country, particularly for tokophobia. In a logistic regression multivariate model, TSH concentration during the first trimester appeared to influence the risk of miscarriage (OR = 1.22, CI95 [1.03–1.65]; *p* = 0.045). An ROC analysis revealed that the optimal cut-off of TSH was 1.55 mIU/L, although its predictive value was low. In a multivariate analysis, TgAbs during the first trimester were one of the determinants of preeclampsia (OR 1.13, CI95 [1.04–1.27]; *p* = 0.007). In a univariate model, TgAbs during the first trimester also appeared to impact the risk of gestational hypertension (OR 1.09, CI95 [1.01–1.29]; *p* = 0.024). Moreover, an ROC analysis confirmed that TgAbs during the first trimester had a predictive value for gestational hypertension (AUC = 0.6, CI95 [0.439–0.768]; *p* = 0.026; PPV = 0.33; NPV = 0.96; optimal cut-off of TgAbs, 94.85 IU/mL).

Gestational hypertension and preeclampsia belong to hypertensive disorders of pregnancy (HDP). Although the etiology of HDP is complex and not fully elucidated, one of its mechanisms, specifically that of early-onset preeclampsia (before 34 weeks of gestation), is defective trophoblast invasion and the incomplete remodeling of spiral arteries, leading to poor uteroplacental perfusion [51]. Consequently, several pro-inflammatory and anti-angiogenic factors are released into the maternal circulation, leading to systemic endothelial dysfunction.

In our TPOAb-/TgAb-positive group, we observed the highest concentration of anti-angiogenic sEng during all three trimesters, although significant differences were only observed with antibody-negative hypothyroid women. In addition, we observed several detrimental correlations between thyroid antibodies and placental angiogenic factors, as follows: a positive correlation between TgAbs and the sEng/PlGF ratio during the first trimester, a positive correlation between TgAbs and the sFlt-1/PlGF ratio during the third trimester, and a positive correlation between TPOAbs and anti-angiogenic sFlt-1 during the third trimester. We also found a negative correlation between TgAbs and pro-angiogenic PlGF during the first trimester and a negative correlation between TgAbs and estradiol during the third trimester.

TPOAb-/TgAb-positive hypothyroid pregnant women had lower estradiol concentrations in the first trimester of pregnancy than antibody-negative hypothyroid women and lower progesterone concentrations in the second trimester than healthy controls. Together, these results provide evidence that thyroid antibodies may be involved in the disturbance of the placentation process and placental function.

For a long time, the pathogenic role of TgAbs in female reproductive function, in contrast to TPOAbs, was understudied. Despite its negative impact on TSH concentration in pregnant women [25,26], Chen et al. demonstrated that TgAbs are associated with an increased risk of premature ruptures of fetal membranes and low birth weight [52]. Saki et al. observed that TPOAb and TgAb positivity were both linked to a higher risk of preeclampsia, preterm delivery, FGR, and a low first-minute Apgar score, although the association for preeclampsia was only documented in subjects with coexisting thyroid dysfunction [53].

In a recent systematic review and meta-analysis, Huisman et al. demonstrated that women with recurrent pregnancy loss (RPL) were more often TgAb-positive than women without RPL; the OR for TgAb positivity was 1.93 (CI95 [1.27–2.92]; I2 = 63%), compared with women without RPL. However, the association was stronger for TgAbs and/or TPOAbs: OR 2.66 (CI95 [1.75–4.05]; I2 = 69%) [54]. Similar to our results, Han et al. reported a prospective cohort study based on 2883 Chinese pregnant women demonstrating that TgAb positivity in the first trimester was an independent risk factor for HDP (OR 1.78, 95% CI95 [1.16–2.73]), mainly gestational hypertension [55]. This relationship persisted when the analysis was performed among euthyroid TgAb-positive women: OR 1.91 (CI95 [1.20–3.02]). A similar association was shown for TPOAb positivity during the first and second trimesters. However, the authors did not find any relationship between thyroid antibodies and the risk of preeclampsia. In a recent multicenter retrospective study comprising 27,408 Chinese pregnant women with 5342 (19.5%) TAI participants, positive thyroid antibodies were independently associated with higher risks of gestational hypertension (OR 1.215, CI95 [1.026–1.439]), gestational diabetes mellitus (OR 1.088, CI95 [1.001–1.183]), and neonatal admission to the NICU (OR 1.084, CI95 [1.004–1.171]) [56]. The results were adjusted for maternal age, BMI, gravidity, TSH and fT4 concentrations, and history of infertility. The authors also conducted a quantitative analysis showing that TPOAb concentration was correlated with a higher probability of gestational hypertension. Additionally, TgAb concentration was positively correlated with gestational hypertension, SGA, and newborn NICU admission. Both TPOAb and TgAb concentrations were negatively associated with neonatal birth weight.

In an Italian study, the authors investigated placental pathologic features and pregnancy outcomes in women who were positive for TPOAb, taking into consideration TSH ≥ 2.5 mU/L and TSH < 2.5 mU/L groups [57]. They concluded that TPOAb positivity during the first trimester was linked to abnormal placentation and negative maternal and neonatal outcomes, irrespective of TSH concentration. Nevertheless, women who were positive for TPOAb with TSH < 2.5 mU/L had fewer pregnancy complications than those with TSH ≥ 2.5 mU/L. During a pathological examination of the placenta, the authors observed the features of decidual arteriopathy and severe maternal vascular malperfusion. These pathological findings were in line with increased uterine and umbilical artery pulsatility index, as determined by the Doppler technique in the first and second trimesters. Importantly, these findings were also in line with an increased risk of SGA/FGR and an increased risk of preeclampsia. Our results differed from those of the Italian group in several ways. First, we did not demonstrate worse pregnancy outcomes in TPOAb-/TgAb-positive women compared with healthy controls, even when the study group was split into subgroups with TSH < 2.5 mIU/L vs. TSH ≥ 2.5 mIU/L and TSH < 3.0 mIU/L vs. TSH ≥ 3.0 mIU/L. The possible reasons might be as follows: (1.) our control group presented an unexpectantly high rate of pregnancy complications—gestational diabetes, 25.6% (vs. 7.3% in the Italian study), preeclampsia, 2.6% (vs. 0.9% in the Italian study), preterm delivery, 12.8% (vs. 0% in the Italian study); (2.) our study group was treated with LT4 and may have had lower TSH concentrations than subjects in the Italian study; and (3.) the size of our study was too small to demonstrate the real impact of thyroid antibodies on pregnancy outcomes. Second, in contrast to the Italian study, we did not observe any differences in the pulsatility index of the uterine artery between groups. However, higher serum sEng concentrations in our TPOAb-/TgAb-positive group and positive correlations between thyroid antibodies and anti-angiogenic factors indicate that TPOAbs/TgAbs can cause placental hypoperfusion. Moreover, the angiogenic factors can be more sensitive markers of uteroplacental ischemia than hemodynamic measurements.

In our study, a weak relationship between cervical insufficiency and TPOAb positivity in the second trimester was found using a univariate regression analysis (OR 1.01, CI95 [1.00–1.03]; *p* = 0.041). A relationship was also observed in a subsequent ROC analysis (AUC 0.703, CI95 [0.504–0.863]; *p* = 0.038). This association may be accidental, as the well-known causes of cervical insufficiency include anatomic abnormality, collagen disorders, obstetric trauma, and previous gynecologic procedures with mechanical cervix dilatation. However, in a recent study, Jeong et al. demonstrated the overexpression of several soluble immune-checkpoint proteins (CD 28, TIM-3, LAG-3, PD-1, and PD-L2) and inflammatory cytokines (CCL2, CCL3, CCL4, IL-6, and TNF-α) in the endocervical microenvironments in patients with cervical insufficiency and preterm delivery [58]. Dysregulation of TIM-3 expression has been associated with excessive or inhibited inflammatory responses, leading to autoimmune disease and pregnancy complications, while TNF-α and IL-6 have a well-documented role in TAI pathogenesis. Hence, TAI and cervical insufficiency may share some common pathological pathways, although further investigations are needed.

It should be noted that several studies have reported no relationship between TAI and negative pregnancy outcomes. In the Northern Finland Birth Cohort Study, with 5805 participants, serum samples were collected before the 20th gestational week, and no association between thyroid antibodies (TPOAb or TgAb) and the risk of preeclampsia and diabetes mellitus was found [59]. No relationship between TPOAb positivity in the first trimester and gestational hypertension or preeclampsia was found in another large prospective cohort study of 5153 women in Rotterdam, the Netherlands [60]. Additional studies on the influence of thyroid antibodies, mainly TPOAbs, on placental morphology are limited, and their results are conflicting. Männistö et al., based on the Northern Finland Birth Cohort, demonstrated higher placental weights in women with TPOAb positivity and hypothyroid mothers [61]. In contrast, other authors have found a negative relationship between TPOAb positivity and placental weights and measurements, including length, width, area, and volume [57,62,63].

Our study was not aimed at assessing the value of selected angiogenic factors for predicting pregnancy outcomes, and the importance of the sFlt-1/PlGF ratio in predicting preeclampsia is well-established [64,65]. However, we found that sEng in the second trimester was a valuable biomarker of preterm delivery (OR 1.45, CI95 [1.00–2.13]; *p* = 0.046) and gestational hypertension (OR 1.91, CI95 [1.16–3.48]; *p* = 0.016) and that the sEng/PlGF ratio in the second trimester was a good predictor of preeclampsia (OR 19.85, CI95 [1.36–879.73]; *p* = 0.040). An ROC analysis demonstrated that sFlt-1 and the sFlt-1/PlGF ratio were only good predictors of gestational hypertension in the third trimester. This observation suggests that sEng serum concentration can be used in clinical practice as an early indicator of possible pregnancy complications.

The strength of our study lies in its prospective character, which permitted the strict monitoring of the participants every four weeks throughout pregnancy, the clinical significance associated with a high prevalence of thyroid autoimmunity in women of reproductive age, and the originality of the topic. To the best of our knowledge, this is the first study to investigate the impact of thyroid antibodies on placental function by accessing its angiogenic factors. An increase in serum anti-angiogenic factors may precede an increase in uterine artery pulsatility index assessed using the Doppler technique. Our study also identified the significance of TgAbs determination in the prediction of pregnancy negative outcomes, although this association should be confirmed in well-controlled studies before it can be translated into clinical practice. We also identified several limitations of our study, most importantly, the small size of the groups. This was mainly due to the fact that our investigation was conducted during the COVID-19 pandemic. Another limitation is the unrepresentative character of the control group (Group 3), which demonstrated a high rate of pregnancy complications. While the exclusion criteria we adopted eliminated participants with chronic illnesses from this study (and Group 3 did not differ from the other groups with regard to its general characteristics), family history and genetic burden were not taken into account. Finally, we could not analyze the Doppler hemodynamic measurements taken at 11–14 weeks of gestation due to incomplete data. In our opinion, more prospective cohort studies are needed to elucidate the influence of thyroid antibodies, especially TgAbs, on pregnancy outcomes. Further research should explore whether TgAbs exert their harmful effect on the pregnancy course independently or via the potentiation of TPOAb action. Also, more studies on the possible expression of thyroid proteins, including thyroglobulin and thyroperoxidase, in female reproductive organs and placenta are necessary to provide an insight into the pathophysiological pathways of thyroid antibodies.

## 5. Conclusions

This study demonstrates that thyroid antibodies, including TgAbs, can be a risk factor for gestational hypertension and preeclampsia in women with chronic lymphocytic thyroiditis. Our observations suggest that TPOAbs and TgAbs can exert a direct harmful effect on the placentation process with a consequent disturbance in the production of placental angiogenic factors. Because of the small size of our study and the scarcity of studies from other authors, further investigations on the negative role and mechanism of action of thyroid antibodies on pregnancy outcomes are needed. However, based on our present results, both thyroid antibodies, TPOAbs and TgAbs, should be included in a diagnostic workshop for women with high-risk pregnancies.

## Figures and Tables

**Table 1:** Clinical characteristics and thyroid function tests of the participants.

Variable	Group 1	Group 2	Group 3 (Controls)	*p*
	N = 58	N = 33	N = 39	
Age, years				0.294
M ± SD	30.74 ± 4.53	28.95 ± 5.05	29.77 ± 4.59	
Weeks of gestation at enrollment				0.439
M ± SD	9.78 ± 2.22	10.32 ± 2.33	9.54 ± 1.96	
BMI, kg/m^2^				0.995
M ± SD	23.70 ± 4.24	25.26 ± 8.26	23.41 ± 4.06	
Education, n (%)				
Higher	43 (74.1)	24 (72.7)	27 (69.2)	0.843
Secondary	14 (24.1)	8 (24.3)	11 (28.2)	
Primary	1 (1.8)	1 (3.0)	1 (2.6)	
Smoking, n (%)				
Yes	1 (1.7)	0 (0.0)	1 (2.6)	
No	57 (98.3)	33 (100.0)	38 (97.4)	>0.999
Parity, n (%)				
Primiparous	29 (50.0)	18 (57.6)	23 (59.0)	0.646
Multiparous	29 (50.0)	14 (42.4)	16 (41.0)	
1st trimester				
TSH				
M ± SD	2.95 ± 3.87	1.06 ± 0.57	1.72 ± 0.96	<0.001
Me (Q1;Q3)	2.08 (1.35;3.10)	1.06 (0.60;1.52)	1.56 (0.99;2.34)	
fT4				
M ± SD	17.09 ± 2.63	15.92 ± 1.85	16.48 ± 3.09	0.053
Me (Q1;Q3)	17.04 (15.47;18.66)	15.82 (14.70;17.09)	16.96 (14.79;18.72)	
fT3				
M ± SD	3.02 ± 0.36	3.20 ± 0.26	3.02 ± 0.32	0.021
Me (Q1;Q3)	2.96 (2.81;3.27)	3.18 (3.06;3.33)	2.98 (2.84;3.22)	
TPOAbs				
M ± SD	932.42 ± 503.5	30.34 ± 5.98	32.38 ± 6.49	<0.001
Me (Q1;Q3)	1300.0 (414.2;1300.0)	28.0 (28.0;28.0)	28.0 (28.0;36.2)	
TgAbs				
M ± SD	41.87 ± 80.3	15.0 ± 0.0	15.0 ± 0.0	<0.001
Me (Q1;Q3)	15.0 (15.0;16.58)	15.0 (15.0;15.0)	15.0 (15.0;15.0)	
CRP				
M ± SD	3.26 ± 3.06	3.58 ± 3.62	4.00 ± 5.93	
Me (Q1;Q3)	2.83 (1.01;4.05)	1.98 (1.02;5.10)	1.93 (1.11;3.96)	0.955
2nd trimester				
TSH				
M ± SD	1.44 ± 0.81	1.43 ± 0.50	1.68 ± 0.58	0.114
Me (Q1;Q3)	1.38 (0.79;1.72)	1.48 (1.16;1.75)	1.75 (1.44;2.04)	
fT4				
M ± SD	15.97 ± 2.35	13.61 ± 1.86	14.99 ± 1.93	<0.001
Me (Q1;Q3)	16.05 (14.39;16.93)	13.46 (12.18;14.93)	15.62 (13.31;16.59)	
fT3				
M ± SD	2.79 ± 0.31	2.78 ± 0.28	2.69 ± 0.24	0.226
Me (Q1;Q3)	2.74 (2.58;2.92)	2.80 (2.67;2.95)	2.65 (2.54;2.80)	
TPOAbs				
M ± SD	806.96 ± 553.11	32.05± 9.6	32.0 ± 7.41	<0.001
Me (Q1;Q3)	1300.0 (217.8;1300.0)	28.0 (28.0;28.0)	28.0 (28.0;32.7)	
TgAbs				
M ± SD	32.95 ± 69.54	15.0 ± 0.0	15.0 ± 0.0	<0.001
Me (Q1;Q3)	15.0 (15.0;25.48)	15.0 (15.0;15.0)	15.0 (15.0;15.0)	
CRP				
M ± SD	4.52 ± 4.08	3.59 ± 3.15	4.32 ± 5.35	
Me (Q1;Q3)	3.18 (1.92;5.11)	2.37 (1.55;4.78)	3.37 (1.83;4.74)	0.654
3rd trimester				
TSH				
M ± SD	1.11 ± 0.63	1.47 ± 0.67	1.08 ± 0.50	0.033
Me (Q1;Q3)	1.02 (0.68;1.44)	1.30 (1.08;1.88)	1.17 (0.68;1.27)	
fT4				
M ± SD	15.70 ± 2.24	13.54 ± 1.61	15.09 ± 1.79	<0.001
Me (Q1;Q3)	15.48 (14.18;17.15)	13.51 (12.25;14.92)	14.82 (14.12;15.91)	
fT3				
M ± SD	2.71 ± 0.36	2.74 ± 0.28	2.73 ± 0.32	0.755
Me (Q1;Q3)	2.66 (2.45;2.84)	2.67 (2.51;2.97)	2.62 (2.52;2.85)	
TPOAbs				
M ± SD	661.19 ± 562.48	29.85 ± 4.65	30.93 ± 7.63	<0.001
Me (Q1;Q3)	397.05 (116.45;1300.0)	28.0 (28.0;28.0)	28.0 (28.0;28.0)	
TgAbs				
M ± SD	31.54 ± 70.76	15.0 ± 0.0	15.0 ± 0.0	<0.001
Me (Q1;Q3)	15.00 (15.0;17.65)	15.0 (15.0;15.0)	15.0 (15.0;15.0)	
CRP				
M ± SD	3.93 ± 3.69	3.29 ± 1.99	3.28 ± 2.27	
Me (Q1;Q3)	2.79 (2.07;4.34)	2.93 (1.69;4.55)	2.54 (1.73;3.94)	0.865

M–mean; SD–standard deviation; Me–median; Q1–1st quartile; Q3–3rd quartile. BMI–body mass index; TSH–thyroid-stimulating hormone; fT4–free thyroxine; fT3–free triiodothyronine TPOAbs–thyroid peroxidase antibodies; TgAbs–thyroglobulin antibodies; CRP–C-reactive protein.

**Table 2:** Obstetrical and neonatal outcomes in study groups.

Variable	Group 1	Group 2	Group 3 (Controls)	*p*
Miscarriage, n (%)	4 (6.9)	0 (0.0)	3 (7.7)	0.317
Preterm birth, n (%)	5 (8.6)	1 (3.0)	5 (12.8)	0.339
Cervical insufficiency, n (%)	6 (10.3)	1 (3.0)	1 (2.6)	0.321
Gestational diabetes, n (%)	10 (17.2)	3 (9.1)	10 (25.6)	0.185
Gestational hypertension, n (%)	3 (5.2)	0 (0.0)	4 (10.3)	0.196
Preeclampsia, n (%)	2 (3.4)	0 (0.0)	1 (2.6)	0.791
Weeks of gestation at birth, Me (Q1;Q3)	39.00 (38.00;40.00)	39.00 (38.00;40.00)	39.00 (38.00;40.00)	0.805
Cesarean section, n (%)	21 (43.8)	6 (33.3)	7 (21.9)	0.130
Birth weight, g				
Males, Me (Q1;Q3)	3435.00 (3197.50;3837.50)	3160.00 (3000.00;3632.50)	3455.00 (3242.50;3747.50)	0.457
Females, M ± SD	3347.75 ± 528.21	3495.00 ± 292.73	3056.25 ± 777.18	0.211
Apgar [0–10], Me (Q1;Q3)	10.00 (10.00;10.00)	10.00 (9.25;10.00)	10.00 (10.00;10.00)	0.288
Apgar below 5, n (%)	0 (0.0)	0 (0.0)	0 (0.0)	-
NICU stay of newborns, n (%)	0 (0.0)	0 (0.0)	0 (0.0)	-
SGA/FGR, n (%)	1 (1.7)	0 (0.0)	0 (0.0)	>0.999

M–mean, SD–standard deviation, Me–median, Q1–1st quartile, Q3–3rd quartile, NICU–neonatal intensive care unit; SGA–small for gestational age; FGR–fetal growth restriction.

**Table 3:** Placental hormones and angiogenic factors.

Variable	Group 1	Group 2	Group 3 (Controls)	Pairwise Comparisons (*p* adj)			
				*p*	Group 1 vs. Group 2	Group 1 vs. Group 3	Group 2 vs. Group 3
1st trimester							
β-hCG	82,601.50 (48,965.00;109,802.50)	77,257.00 (57,513.00;137,203.00)	90,483.00 (69,269.50;107,376.50)	0.338	-	-	-
P	23.14 ± 9.28	24.13 ± 7.73	24.30 ± 9.56	0.792	-	-	-
E2	1682.00 (1138.25;2560.00)	2440.00 (1820.00;4420.00)	1448.00 (1130.00;2700.00)	0.008	0.016	> 0.999	0.017
PlGF	25.95 (13.65;42.63)	27.40 (15.28;41.03)	25.70 (10.12;35.45)	0.299	-	-	-
sEng	6.79 ± 2.18	5.73 ± 1.41	5.93 ± 1.49	0.014	0.026	0.065	0.896
sFlt-1	6540.00 (4617.50;8205.00)	5440.00 (3648.00;7353.00)	5675.00 (4379.50;8144.50)	0.263	-	-	-
sEng/PIGF	262.98 (152.99;474.43)	200.17 (134.18;355.98)	233.69 (151.12;577.46)	0.204	-	-	-
sFlt-1/PIGF	224.31 (166.20;403.60)	159.11 (99.84;342.83)	268.89 (164.01;479.17)	0.046	0.157	> 0.999	0.050
2nd trimester							
β-hCG	14,611.50 (10,946.75;25,252.00)	18,545.00 (11,844.50;29,143.00)	15,950.00 (11,975.00;28,136.75)	0.543	-	-	-
P	37.00 (30.05;48.20)	48.90 (37.45;53.40)	50.25 (40.48;57.50)	0.004	0.224	0.003	> 0.999
E2	7800.00 (5980.00;10,780.00)	8760.00 (6950.00;13,150.00)	8480.00 (6460.00;9745.00)	0.495	-	-	-
PlGF	142.45 (83.40;234.80)	176.20 (118.65;214.05)	188.05 (110.58;298.30)	0.338	-	-	-
sEng	5.81 ± 1.76	4.68 ± 0.75	5.16 ± 1.55	0.018	0.022	0.160	0.532
sFlt-1	6415.00 (4244.50;7820.50)	4922.00 (3285.00;6853.00)	5742.00 (4236.00;8204.50)	0.301	-	-	-
sEng/PIGF	38.21 (21.44;68.21)	29.97 (20.50;39.37)	21.68 (13.75;64.33)	0.076	-	-	-
sFlt-1/PIGF	40.94 (24.09;61.34)	25.06 (22.40;45.77)	33.09 (17.98;51.58)	0.289	-	-	-
3rd trimester							
β-hCG	15,259.00 (11,194.00;29,116.00)	14,172.00 (5531.25;27,111.75)	17,554.00 (10,573.00;32,237.00)	0.484	-	-	-
P	97.62 ± 41.24	97.61 ± 32.08	102.84 ± 36.72	0.830	-	-	-
E2	14,070.00 (9897.50;16,849.00)	14,910.00 (13,185.00;17,855.00)	13,860.00 (11,220.00;16,020.00)	0.214	-	-	-
PlGF	378.90 (240.60;768.40)	472.00 (247.45;603.90)	408.60 (229.00;691.00)	0.979	-	-	-
sEng	7.80 (6.90;10.00)	5.60 (4.70;7.38)	6.50 (5.20;8.70)	0.003	0.003	0.110	0.480
sFlt-1	7873.00 (5462.00;11,976.00)	5906.50 (4227.50;8412.00)	7820.00 (5196.00;9712.00)	0.129	-	-	-
sEng/PIGF	21.71 (9.81;36.37)	16.70 (8.79;25.21)	12.44 (8.37;42.25)	0.490	-	-	-
sFlt-1/PIGF	17.49 (11.40;42.34)	16.48 (9.27;24.00)	17.02 (9.90;45.97)	0.562	-	-	-

β-hCG–beta-human chorionic gonadotropin; P–progesterone; E2–estradiol; PlGF–placental growth factor; sEng–soluble endoglin; sFlt-1–soluble FMS-like tyrosine kinase-1.

**Table 4:** Correlations among TPOAbs, TgAbs and placental hormones, angiogenic factors, and hemodynamic parameters.

	TPOAbs		TgAbs	
	rho	*p*	rho	*p*
Placental hormones–1st trimester				
β-hCG	−0.02	0.903	0.13	0.335
P	−0.07	0.600	−0.21	0.117
E2	0.07	0.581	−0.20	0.132
Placental hormones–2nd trimester				
β-hCG	0.01	0.938	0.17	0.233
P	0.03	0.815	0.08	0.561
E2	−0.05	0.742	−0.15	0.286
Placental hormones–3rd trimester				
β-hCG	−0.13	0.363	0.05	0.752
P	−0.02	0.896	−0.09	0.556
E2	0.13	0.393	−0.29	0.043
Placental angiogenic factors–1st trimester				
PlGF	0.09	0.508	−0.27	0.037
sEng	−0.04	0.772	0.11	0.409
sFlt-1	−0.06	0.668	−0.10	0.445
sEng/PIGF	−0.08	0.563	0.28	0.034
sFlt-1/PIGF	−0.04	0.779	0.22	0.105
Placental angiogenic factors–2nd trimester				
PlGF	−0.03	0.821	0.00	0.977
sEng	−0.02	0.878	0.19	0.175
sFlt-1	0.06	0.692	−0.05	0.753
sEng/PIGF	0.01	0.971	0.07	0.642
sFlt-1/PIGF	0.14	0.316	−0.11	0.432
Placental angiogenic factors–3rd trimester				
PlGF	−0.18	0.243	−0.21	0.165
sEng	0.05	0.736	0.19	0.200
sFlt-1	0.31	0.040	0.20	0.182
sEng/PIGF	0.15	0.324	0.24	0.109
sFlt-1/PIGF	0.24	0.114	0.30	0.049
Placental hemodynamics 18–22 WG				
UA-PI	0.16	0.285	−0.16	0.275
UtA-RI right	0.13	0.398	−0.05	0.754
UtA-RI left	−0.01	0.921	−0.03	0.863
UtA-PI right	0.23	0.122	−0.08	0.581
UtA-PI left	−0.06	0.670	0.07	0.628
Placental hemodynamics 28–32 WG				
UA-PI	0.04	0.785	0.16	0.277
UtA-RI right	0.24	0.101	0.13	0.368
UtA-RI left	0.07	0.640	0.01	0.967
UtA-PI right	0.20	0.168	0.19	0.200
UtA-PI left	0.08	0.601	−0.02	0.873

TPOAb–thyroid peroxidase antibody; TgAb–thyroglobulin antibody; β-hCG–beta-human chorionic gonadotropin; P–progesterone; E2–estradiol; PlGF–placental growth factor; sEng–soluble endoglin; sFlt-1–soluble FMS-like tyrosine kinase-1; WG–weeks of gestation; UA-PI–umbilical artery pulsatility index; UtA-RI–uterine artery resistance index; UtA-PI–uterine artery pulsatility index; rho–Spearman correlation coefficient.

**Table 5:** Multivariate logistic regression model of factors that are potentially predictive of pregnancy complications.

Variable	1st Trimester		2nd Trimester		3rd Trimester	
	OR (95% CI)	*p*	OR (95% CI)	*p*	OR (95% CI)	*p*
Model for miscarriage						
Age, years	1.18 (0.97;1.50)	0.132	-	-	-	-
TSH	1.22 (1.03;1.65)	0.045	-	-	-	-
PlGF	1.02 (1.00;1.05)	0.076	-	-	-	-
Model for preterm birth						
TgAbs in 10 units	1.07 (0.99;1.16)	0.063	1.09 (1.00;1.31)	0.100	-	-
PlGF	-	-	-	-	1.00 (0.99;1.00)	0.207
sEng	-	-	1.45 (1.00;2.13)	0.046	1.23 (0.94;1.73)	0.155
sFlt-1 in 1000 units	0.80 (0.60;1.01)	0.089	-	-	1.28 (1.05;1.65)	0.025
sFlt-1/PlGF in 100 units	-	-	-	-	0.10 (0.00;1.15)	0.230
Model for cervical insufficiency						
Parity (multiparous vs. primiparous)	-	-			0.32 (0.05;1.52)	0.187
TPOAbs in 10 units	1.01 (1.00;1.02)	0.126	1.00 (1.00;1.00)	0.060	1.01 (1.00;1.02)	0.170
sEng	-	-	1.38 (0.88;2.14)	0.144	-	-
sEng/PIGF in 100 units	1.11 (1.03;1.21)	0.010	-	-	-	-
Model for gestational hypertension						
Parity (multiparous vs. primiparous)	0.23 (0.01;1.51)	0.188	0.21 (0.01;1.55)	0.185	0.07 (0.00;1.09)	0.125
TgAbs in 10 units	1.07 (1.00;1.18)	0.060	1.12 (1.02;1.43)	0.086	-	-
sEng	-	-	1.91 (1.16;3.48)	0.016	1.37 (1.05;1.91)	0.028
sFlt-1/PIGF in 100 units	-	-	-	-	7.49 (1.57;71.74)	0.039
Model for preeclampsia						
Parity (multiparous vs. primiparous)						
TgAbs in 10 units	1.13 (1.04;1.27)	0.007	1.37 (1.06;2.45)	0.281	-	-
sEng	1.57 (0.82;3.09)	0.143	-	-	-	-
sEng/PIGF in 100 units	-	-	19.85 (1.36;879.73)	0.040	-	-

OR–odds ratio, CI–confidence interval; BMI–body mass index; TSH–thyroid-stimulating hormone; TPOAbs–thyroid peroxidase antibodies; TgAbs–thyroglobulin antibodies; PlGF–placental growth factor; sEng–soluble endoglin; sFlt-1–soluble FMS-like tyrosine kinase-1.

**Table 6:** Receiver operating characteristics (ROC) analysis for selected pregnancy complications.

Complication	Cut-Off	AUC (95% CI)	Sensitivity	Specificity	Accuracy	PPV	NPV	*p*
Miscarriage								
TSH (1st trimester)	1.55	0.549 (0.357;0.745)	0.71	0.52	0.53	0.08	0.97	0.037
Preterm birth								
sEng (2nd trimester)	5.45	0.706 (0.552;0.837)	0.73	0.66	0.66	0.21	0.95	0.058
sEng (3rd trimester)	9.25	0.698 (0.471;0.879)	0.60	0.80	0.78	0.27	0.94	0.001
s-Flt-1 (3rd trimester)	8798.00	0.769 (0.601;0.915)	0.80	0.70	0.71	0.24	0.97	0.002
sEng/PIGF (3rd trimester)	28.88	0.707 (0.521;0.861)	0.70	0.71	0.71	0.23	0.95	0.815
Cervical insufficiency								
TPOAbs (2nd trimester)	1143.85	0.703 (0.504;0.863)	0.62	0.77	0.75	0.19	0.96	0.038
sEng/PIGF (1st trimester)	214.22	0.673 (0.439;0.875)	0.88	0.50	0.52	0.10	0.98	0.005
s-Flt-1/PIGF (1st trimester)	349.69	0.643 (0.384;0.876)	0.75	0.68	0.69	0.14	0.98	0.011
Gestational hypertension								
TgAbs (1st trimester)	94.85	0.600 (0.439;0.768)	0.29	0.97	0.93	0.33	0.96	0.026
sEng (2nd trimester)	5.35	0.765 (0.615;0.904)	0.86	0.63	0.64	0.15	0.98	0.019
sEng (3rd trimester)	7.05	0.778 (0.599;0.924)	1.00	0.51	0.54	0.12	1.00	0.001
PIGF (3rd trimester)	283.25	0.857 (0.733;0.957)	1.00	0.70	0.72	0.19	1.00	0.002
s-Flt-1(3rd trimester)	17,162.00	0.797 (0.486;0.996)	0.67	0.97	0.95	0.57	0.98	<0.001
sEng/PIGF (2nd trimester)	43.17	0.728 (0.474;0.912)	0.86	0.68	0.69	0.17	0.98	0.061
s-Flt-1/PIGF (3rd trimester)	72.18	0.882 (0.702;0.988)	0.83	0.91	0.90	0.38	0.99	<0.001

AUC–area under curve; CI–confidence interval; PPV–positive predictive value; NPV–negative predictive value; TSH–thyroid-stimulating hormone; TPOAbs–thyroid peroxidase antibodies; TgAbs–thyroglobulin antibodies; PlGF–placental growth factor; sEng–soluble endoglin; sFlt-1–soluble FMS-like tyrosine kinase-1.

## Data Availability

The original contributions presented in this study are included in the article/Supplementary Materials; further inquiries can be directed to the corresponding authors.

## Supplementary Materials

The following supporting information can be downloaded at https://www.mdpi.com/article/10.3390/biomedicines12112628/s1: Supplementary Table S1: Obstetrical and neonatal outcomes regarding different TSH concentrations: below and equal to/over 2.5 mIU/L; Supplementary Table S2: Obstetrical and neonatal outcomes regarding different TSH concentrations: below and equal to/over 3.0 mIU/L; Supplementary Table S3: Comparison of placental hemodynamics between study groups; Supplementary Table S4: Univariate logistic regression model of factors that are potentially predictive of pregnancy complications.
