# Supplementary material for: Antithyroglobulin and Antiperoxidase Antibodies Can Negatively Influence Pregnancy Outcomes by Disturbing the Placentation Process and Triggering an Imbalance in Placental Angiogenic Factors
Source: Biomedicines. 2024 Nov 17;12(11):2628. doi: 10.3390/biomedicines12112628 (PMC11592358; doi:10.3390/biomedicines12112628)
Supplement: Supplementary file 1 [file biomedicines-12-02628-s001.zip › Supplementary Table S1.pdf]

Supplementary Material

Table S1. Obstetrical and neonatal outcomes regarding different TSH concentrations: below- and equal/over 2.5 mIU/L

Variable	Group 1 (TSH < 2.5 mIU/L)	Group 1 (TSH ≥ 2.5 mIU/L)	Group 2	Group 3 (Controls)	*p*
Miscarriage, *n* (%)	3 (8.3)	1 (4.5)	0 (0.0)	3 (7.7)	0.382
Preterm birth, *n* (%)	4 (11.1)	1 (4.5)	1 (3.0)	5 (12.8)	0.444
Cervical insufficiency, *n* (%)	4 (11.1)	2 (9.1)	1 (3.0)	1 (2.6)	0.353
Gestational diabetes, *n* (%)	6 (16.7)	4 (18.2)	3 (9.1)	10 (25.6)	0.334
Gestational hypertension, *n* (%)	2 (5.6)	1 (4.5)	0 (0.0)	4 (10.3)	0.286
Preeclampsia, *n* (%)	1 (2.8)	1 (4.5)	0 (0.0)	1 (2.6)	0.870
Weeks of gestation at birth, Me (Q1;Q3)	39.00 (38.00;40.00)	39.00 (38.00;40.00)	39.00 (38.00;40.00)	39.00 (38.00;40.00)	0.900
Cesarean section, *n* (%)	15 (53.6)	6 (30.0)	6 (33.3)	7 (21.9)	0.074
Birth weight, g					
Males, Me (Q1;Q3)	3465.00 (3295.00;3872.50)	3390.00 (3192.50;3745.00)	3160.00 (3000.00;3632.50)	3455.00 (3242.50;3747.50)	0.617
Females, M ± SD	3438.21 ± 580.67	3136.67 ± 327.33	3495.00 ± 292.73	3056.25 ± 777.18	0.251
Apgar [0-10], Me (Q1;Q3)	10.00 (10.00;10.00)	10.00 (10.00;10.00)	10.00 (9.25;10.00)	10.00 (10.00;10.00)	0.147
Apgar below 5, *n* (%)	0 (0.0)	0 (0.0)	0 (0.0)	0 (0.0)	-
NICU stay of newborns, *n* (%)	0 (0.0)	0 (0.0)	0 (0.0)	0 (0.0)	-
SGA/FGR, *n* (%)	1 (1.7)	0 (0.0)	0 (0.0)	0 (0.0)	>0.999

M – mean, SD – standard deviation, Me – median, Q1 – 1st quartile, Q3 – 3rd quartile, NICU- neonatal intensive care unit; SGA- small for gestational age; FGR- fetal growth restriction
