# Supplementary material for: Antithyroglobulin and Antiperoxidase Antibodies Can Negatively Influence Pregnancy Outcomes by Disturbing the Placentation Process and Triggering an Imbalance in Placental Angiogenic Factors
Source: Biomedicines. 2024 Nov 17;12(11):2628. doi: 10.3390/biomedicines12112628 (PMC11592358; doi:10.3390/biomedicines12112628)
Supplement: Supplementary file 1 [file biomedicines-12-02628-s001.zip › Supplementary Table S3.pdf]

Supplementary Material

Table S3. Comparison of placental hemodynamics between study groups.

**Variable**	**Group 1**	**Group 2**	**Group 3 (Controls)**	***p***
Placental hemodynamics 19-21 WG				
UA- PI	1.21 (1.10;1.31)	1.20 (1.10;1.23)	1.21 (1.14;1.27)	0.636
UtA-RI right	0.56 (0.53;0.65)	0.54 (0.48;0.55)	0.64 (0.50;0.70)	0.157
UtA- RI left	0.58 (0.54;0.66)	0.61 (0.54;0.63)	0.58 (0.52;0.70)	0.971
UtA- PI right	0.89 (0.77;1.19)	0.87 (0.80;1.16)	1.10 (0.77;1.29)	0.560
UtA- PI left	1.04 ± 0.34	1.00 ± 0.20	1.08 ± 0.29	0.695
NOTCH, no	47 (100.0)	18 (100.0)	30 (100.0)	-
Placental hemodynamics 31-33 WG				
UA- PI	0.97 (0.85;1.08)	0.96 (0.90;1.07)	1.00 (0.88;1.07)	0.893
UtA-RI right	0.51 ± 0.10	0.50 ± 0.10	0.50 ± 0.09	0.982
UtA- RI left	0.52 ± 0.11	0.48 ± 0.06	0.50 ± 0.09	0.240
UtA- PI right	0.76 (0.64;0.96)	0.69 (0.60;0.88)	0.82 (0.60;0.89)	0.725
UtA- PI left	0.86 (0.61;1.01)	0.72 (0.64;0.81)	0.75 (0.64;0.80)	0.407
NOTCH, no	47 (100.0)	18 (100.0)	29 (100.0)	-

WG- weeks of gestation; UA- PI- umbilical artery pulsatility index; UtA-RI- uterine artery resistance index; UtA-PI- uterine artery pulsatility index.
