# Supplementary material for: Antithyroglobulin and Antiperoxidase Antibodies Can Negatively Influence Pregnancy Outcomes by Disturbing the Placentation Process and Triggering an Imbalance in Placental Angiogenic Factors
Source: Biomedicines. 2024 Nov 17;12(11):2628. doi: 10.3390/biomedicines12112628 (PMC11592358; doi:10.3390/biomedicines12112628)
Supplement: Supplementary file 1 [file biomedicines-12-02628-s001.zip › Supplementary Table S4.pdf]

Supplementary Material

Table S4. Univariate logistic regression model of factors potentially predictive for pregnancy complications.

Variable	General		1st trimester		2nd trimester		3rd trimester	
	OR (95% CI)	*p*	OR (95% CI)	*p*	OR (95% CI)	*p*	OR (95% CI)	*p*
Models for miscarriage								
Age, years	1.12 (0.94;1.37)	0.239	-	-	-	-	-	-
BMI, kg/m^2	1.05 (0.90;1.17)	0.479	-	-	-	-	-	-
Weight gain, kg	0.86 (0.45;1.50)	0.577	-	-	-	-	-	-
Parity (multiparous vs primiparous)	0.89 (0.17;4.20)	0.877	-	-	-	-	-	-
TSH	-	-	1.18 (1.01;1.53)	0.060	0.00 (0.00;0.25)	0.215	-	-
FT4	-	-	0.86 (0.63;1.16)	0.344	3.01 (1.25;37.01)	0.096	-	-
TPOAbs in 10 units	-	-	1.01 (0.99;1.02)	0.384	1.62	0.998	-	-
TgAbs in 10 units	-	-	0.00	0.994	0.03 (0.00;7.55)	0.891	-	-
PlGF	-	-	1.02 (0.99;1.04)	0.132	1.00 (0.96;1.01)	0.705	-	-
sEng	-	-	0.67 (0.35;1.10)	0.170	0.92 (0.13;2.82)	0.903	-	-
sFlt-1 in 1000 units	-	-	0.77 (0.54;1.02)	0.109	1.03 (0.47;1.54)	0.910	-	-
sEng/PIGF in 100 units	-	-	1.00 (0.81;1.10)	0.990	0.48 (0.00;35.92)	0.825	-	-
sFlt-1/PIGF in 100 units	-	-	0.96 (0.69;1.12)	0.703	1.03 (0.00;24.03)	0.990	-	-
Models for preterm birth								
Age, years	1.03 (0.90;1.19)	0.698	-	-	-	-	-	-
BMI, kg/m^2	0.90 (0.73;1.05)	0.268	-	-	-	-	-	-
Weight gain, kg	0.98 (0.83;1.16)	0.808	-	-	-	-	-	-
Parity (multiparous vs primiparous)	0.65 (0.16;2.30)	0.516	-	-	-	-	-	-
TSH	-	-	0.82 (0.43;1.13)	0.456	0.88 (0.32;2.13)	0.789	1.38 (0.49;3.70)	0.529
FT4	-	-	1.10 (0.87;1.38)	0.427	1.18 (0.91;1.53)	0.204	1.03 (0.75;1.38)	0.845
TPOAbs in 10 units	-	-	1.00 (0.99;1.01)	0.621	1.00 (0.99;1.01)	0.548	1.00 (0.99;1.02)	0.495
TgAbs in 10 units	-	-	1.06 (0.98;1.14)	0.099	1.09 (0.99;1.34)	0.149	1.10 (1.00;1.47)	0.182
PlGF	-	-	1.00 (0.97;1.02)	0.974	1.00 (1.00;1.00)	0.966	1.00 (0.99;1.00)	0.113

Variable	General		1st trimester		2nd trimester		3rd trimester	
	OR (95% CI)	*p*	OR (95% CI)	*p*	OR (95% CI)	*p*	OR (95% CI)	*p*
sEng	-	-	1.15 (0.83;1.55)	0.359	1.42 (0.99;2.04)	0.055	1.42 (1.14;1.91)	0.006
sFlt-1 in 1000 units	-	-	0.83 (0.64;1.02)	0.115	0.96 (0.77;1.14)	0.657	1.22 (1.08;1.41)	0.002
sEng/PIGF in 100 units	-	-	1.00 (0.85;1.09)	0.968	1.71 (0.33;6.73)	0.468	0.98	0.840
sFlt-1/PIGF in 100 units	-	-	0.89 (0.63;1.06)	0.360	0.80 (0.10;3.17)	0.789	1.94 (0.68;5.12)	0.162
Models for cervical insufficiency								
Age, years	0.99 (0.85;1.16)	0.876	-	-	-	-	-	-
BMI, kg/m²	0.89 (0.68;1.06)	0.292	-	-	-	-	-	-
Weight gain, kg	0.96 (0.78;1.17)	0.646	-	-	-	-	-	-
Parity (multiparous (vs primiparous)	0.37 (0.05;1.70)	0.239	-	-	-	-	-	-
TSH	-	-	1.06 (0.81;1.24)	0.487	0.59 (0.17;1.74)	0.370	0.56 (0.14;1.83)	0.374
FT4	-	-	0.94 (0.70;1.23)	0.647	1.27 (0.95;1.71)	0.108	1.20 (0.86;1.66)	0.272
TPOAbs in 10 units	-	-	1.01 (1.00;1.03)	0.060	1.01 (1.00;1.03)	0.041	1.01 (1.00;1.02)	0.187
TgAbs in 10 units	-	-	1.01 (0.80;1.10)	0.892	1.01	0.928	0.99	0.935
PlGF	-	-	0.98 (0.93;1.01)	0.360	1.00 (0.99;1.00)	0.981	1.00 (1.00;1.00)	0.452
sEng	-	-	1.14 (0.78;1.59)	0.464	1.41 (0.93;2.12)	0.097	0.99 (0.73;1.24)	0.969
sFlt-1 in 1000 units	-	-	0.99 (0.78;1.20)	0.897	1.14 (0.95;1.34)	0.133	0.99 (0.83;1.15)	0.929
sEng/PIGF in 100 units	-	-	1.12 (1.04;1.22)	0.004	0.97 (0.10;5.14)	0.979	0.27 (0.00;0.87)	0.420
sFlt-1/PIGF in 100 units	-	-	1.15 (1.04;1.28)	0.006	1.19 (0.15;4.77)	0.835	0.25 (0.00;1.86)	0.385
Models for gestational hypertension								
Age, years	1.03 (0.88;1.24)	0.727	-	-	-	-	-	-
BMI, kg/m²	0.97 (0.78;1.12)	0.757	-	-	-	-	-	-
Weight gain, kg	1.08 (0.87;1.36)	0.490	-	-	-	-	-	-
Parity (multiparous vs primiparous)	0.18 (0.01;1.12)	0.121	-	-	-	-	-	-
TSH	-	-	0.78 (0.33;1.15)	0.485	0.79 (0.22;2.33)	0.693	1.67 (0.46;5.68)	0.413
FT4	-	-	0.99 (0.73;1.31)	0.947	0.81 (0.54;1.14)	0.253	1.01 (0.68;1.46)	0.955
TPOAbs in 10 units	-	-	0.99 (0.97;1.01)	0.486	0.99 (0.97;1.01)	0.455	1.00 (0.98;1.01)	0.783
TgAbs in 10 units	-	-	1.09 (1.01;1.20)	0.024	1.14 (1.02;1.58)	0.234	1.15 (1.02;1.79)	0.290

Variable	General		1st trimester		2nd trimester		3rd trimester	
	**OR (95% CI)**	***p***	**OR (95% CI)**	***p***	**OR (95% CI)**	***p***	**OR (95% CI)**	***p***
PlGF	-	-	0.99 (0.95;1.02)	0.629	1.00 (0.99;1.00)	0.745	0.99 (0.98;1.00)	0.031
sEng	-	-	1.36 (0.94;1.93)	0.083	1.68 (1.09;2.67)	0.020	1.47 (1.16;2.05)	0.006
sFlt-1 in 1000 units	-	-	0.90 (0.67;1.13)	0.406	0.95 (0.71;1.17)	0.661	1.33 (1.14;1.64)	0.001
sEng/PIGF in 100 units	-	-	0.99 (0.77;1.10)	0.864	4.65 (0.92;21.65)	0.048	1.00	0.958
sFlt-1/PIGF in 100 units	-	-	0.90 (0.59;1.10)	0.507	2.18 (0.42;8.05)	0.273	9.77 (2.67;54.82)	0.003
Models for preeclampsia								
Age, years	0.94 (0.73;1.20)	0.584	-	-	-	-	-	-
BMI, kg/m^2	1.07 (0.87;1.24)	0.378	-	-	-	-	-	-
Weight gain, kg	0.97 (0.71;1.35)	0.845	-	-	-	-	-	-
Parity (multiparous vs primiparous)	0.00	0.994	-	-	-	-	-	-
TSH	-	-	0.99 (0.40;1.24)	0.976	1.01 (0.16;4.23)	0.994	1.10 (0.09;8.53)	0.930
FT4	-	-	0.97 (0.61;1.46)	0.896	1.09 (0.66;1.70)	0.710	1.77 (0.96;3.94)	0.087
TPOAbs in 10 units	-	-	1.00 (0.98;1.02)	0.775	1.00 (0.98;1.02)	0.777	1.01 (0.99;1.04)	0.323
TgAbs in 10 units	-	-	1.13 (1.04;1.27)	0.007	1.34 (1.05;2.12)	0.241	1.55 (1.07;3.53)	0.291
PlGF	-	-	0.99 (0.92;1.03)	0.811	0.98 (0.95;1.00)	0.189	0.99 (0.97;1.00)	0.192
sEng	-	-	1.46 (0.86;2.42)	0.130	2.23 (1.17;5.19)	0.025	1.10 (0.65;1.48)	0.610
sFlt-1 in 1000 units	-	-	1.04 (0.71;1.38)	0.827	1.09 (0.79;1.38)	0.503	2.60 (1.29;22.42)	0.133
sEng/PIGF in 100 units	-	-	0.95 (0.53;1.12)	0.750	8.23 (0.90;78.98)	0.048	1.00	0.979
sFlt-1/PIGF in 100 units	-	-	0.90 (0.43;1.16)	0.660	5.30 (0.84;29.37)	0.047	9.74 (2.21;152.09)	0.018

OR – odds ratio, CI – confidence interval; BMI- body mass index; TSH- thyroid-stimulating hormone; fT4- free thyroxine; TPOAbs- thyroid peroxidase antibodies; TgAbs- thyroglobulin antibodies; PlGF- placental growth factor; sEng- soluble endoglin; sFlt-1- soluble FMS-like tyrosine kinase-1.
